# Analysis of tangential contact boundary value problems using potential functions

**DOI:** 10.1098/rsos.182106

**Published:** 2019-03-13

**Authors:** Adam G. Taylor, Jae H. Chung

**Affiliations:** Computer Laboratory for Granular Physics Studies, Geosystems Engineering, University of Florida, FL, USA

**Keywords:** Cerruti's problem, elasticity, potential theory, soil–structure interaction, shallow foundation

## Abstract

This paper presents an analysis technique of high-order contact potential problems and its application to an elastic settlement analysis of a shallow foundation system subjected to a combined traction boundary condition. Closed-form solutions of potential functions are derived for an elastic half-space subjected to bilinear tangential traction boundary conditions over rectangular surface regions. Using the principle of superposition, the present solutions provide a means to form an approximate and continuous solution of elastic contact problems with higher-order tangential boundary conditions. As an application example, an elastic settlement analysis of a rigid footing founded on a dense granular soil is performed under a tangential traction boundary condition prescribed in an analogy with the stress equilibrium states of static sandpiles. A generalized solution approach to combined normal and tangential traction boundary value problems is discussed in the context of foundation engineering.

## 1. Introduction

The problem of the equilibrium state of an isotropic elastic half-space with given conditions on its planar boundary has been known historically as ‘the problem of Boussinesq and Cerruti’ [1], after the mathematicians who originally developed solutions to the problem in the late nineteenth century. The solutions to the governing equilibrium equations were given by these authors in terms of potential functions that correspond to specified boundary conditions. The problem where normal tractions are prescribed is often specifically associated with the name of Boussinesq. The tangential traction boundary value problem is then referred to as the Cerruti problem [2,3]. Since these scholars presented both solutions nearly simultaneously [4,5], the distinction appears to be mostly one of convenience. These classical solutions have been widely accepted for use in design of shallow foundations. In shallow foundation systems, contact pressures develop at the interface between the structural footing and a supporting soil body against the external loads transmitted to the soil through the structure. Normal contact pressures are typically prescribed as the traction boundary condition for elastic settlement analysis of shallow foundations subjected to vertically applied concentric loads. The resultants of the contact stresses and the actual distribution in a specific problem are not known *a priori*, and are the final product of the soil–structure interaction, which is statically indeterminate. Under the requirements of static equilibrium, only the average value of the contact pressure is statically determined simply by dividing the averaged resultant load by the area of contact of the (relatively) rigid footing with the soil. In turn, the spatial distribution of the normal traction boundary condition is often simplified to be uniform, and it is assumed that any shear stresses vanish at the contact interface between the footing and a supporting soil medium [6,7]. However, numerous empirical measurements of stresses beneath piles of sand [8–11] and laboratory-scale foundations [12–15] reveal that both normal and shear stresses coexist in a high-order spatial variation that is believed to be dependent on construction and loading histories of the supporting soil and the magnitudes of vertically applied concentric loads. For these reasons, the authors have addressed the problem of calculating the response from highly variable empirical normal traction fields in a past manuscript [16]. Towards the second assumption, it is reasonable that some frictional forces develop in the contact plane where internal resistances of the soil medium may cause confining effects against horizontal expansion when compressed. Thus, it is hypothesized that the tangential traction components may develop even when the footing is subjected to concentric vertical loads. Although there have been few empirical measurements for the shearing stresses beneath vertically loaded shallow foundations (e.g. [13]), a striking similarity between the normal contact pressure distributions of shallow foundations and sandpiles further elaborates the conceptualization of a combined normal and tangential traction boundary condition for shallow foundations. This rationale serves as a motivation for the present study.

The form of the elastic solutions for tangential traction boundary value problems in terms of potential functions is well understood [1]. Yet, closed-form expressions for the potential functions have been calculated only for tractions prescribed by low-order polynomial laws [17,18]. When a traction boundary condition is high order, the calculation of closed-form solutions becomes intractable. It is practical to consider an approximate solution by superimposing known solutions of lower order as per discretization of higher order traction boundary conditions. The conceptual framework of the approximate solution approach [19,20] has shortcomings such that discontinuities in traction fields along the local (discretized) boundaries may lead to singularities in the calculation of stresses when tractions are prescribed over rectangular subdomains in constant and/or unidimensional linear terms only [6]. Recently, progress has been made to overcome these computational difficulties [16,21–24] in obtaining converged solutions for the elastic contact problems.

In an earlier work [16], the authors recommended an approximate yet continuous solution to the problem of high-order normal traction boundary conditions. The present study aims to provide corresponding solutions to tangential traction boundary value problems as a companion to [16]. A complete set of closed-form solutions of potential functions for bilinear tractions and their derivatives is derived through methods of substitution and direct calculation of area integrals. By the interpolation and superposition of these solutions, the displacement and stress fields of the half-space are approximated under combined normal and tangential traction boundary conditions to a desired degree of accuracy. The method is verified through a study of convergence to the classical Hertz–Mindlin contact solution [25,26]. To further the application to foundation design and analysis, a hypothesis is presented to qualitatively prescribe a plausible tangential contact traction field between a rigid square footing and a supporting granular soil. Parameters of empirical expressions are determined in analogy to the stress-equilibrium states of sandpiles [8–11], in particular with respect to the continuum-based fixed principle axis (FPA) model [27,28]. The present method of solution is applied to solving a combined boundary value problem for normal and tangential tractions at the base of a square shallow foundation in a Cartesian coordinate system.

## 2. Description of the boundary value problem

### 2.1. Neumann (traction) boundary conditions

**Figure 1.**
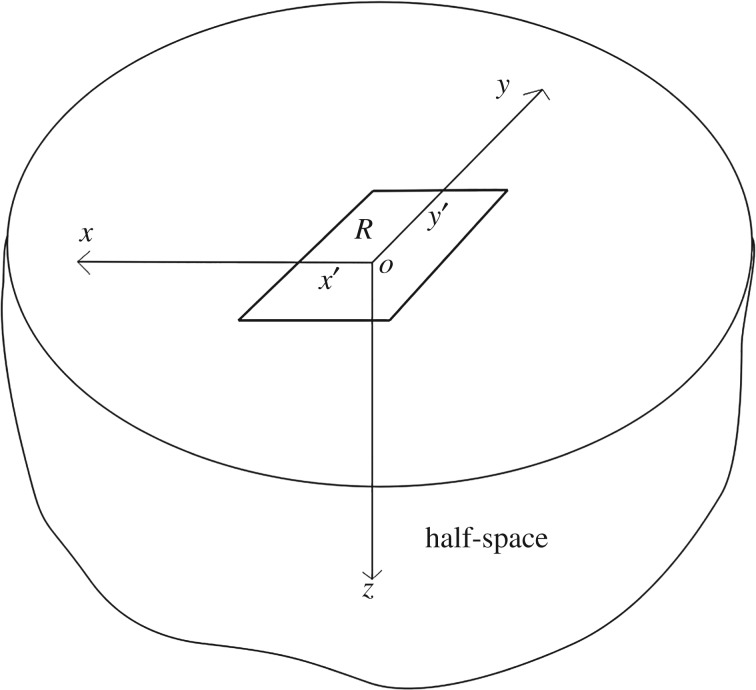
An elastic half-space bounded at *z* = 0 described by a Cartesian coordinate system. This figure is retained from the authors' previous manuscript [16].

Consider a homogeneous, isotropic, linear elastic half-space defined by the set $H=\{(x,\;y,\;z)\in R^{3}|z>0\}$. We define a right-handed set of Cartesian coordinates such that the positive *z*-axis points downwards into the body, as in figure 1. Let the displacement at point (*x*, *y*, *z*) be denoted by (*u*, *v*, *w*). The full details of the boundary value problem are available in many texts on the mathematical theory of elasticity [1,29]. In the Neumann problem, surface traction, given by Cartesian components (*q*_*x*_, *q*_*y*_, *p*), is prescribed on the boundary plane. These expressions for the Cartesian traction boundary conditions are related to the in-plane surface stresses and the partial derivatives of the surface displacements by Hooke's Law

2.1 $$\begin{array}{rl} q_{x} & ={-\sigma_{xz}|}_{z=0}=-\mu \left({\frac{\partial u}{\partial z}|}_{z=0}+{\frac{\partial w}{\partial x}|}_{z=0}\right),\\ q_{y} & ={-\sigma_{yz}|}_{z=0}=-\mu \left({\frac{\partial v}{\partial z}|}_{z=0}+{\frac{\partial w}{\partial y}|}_{z=0}\right)\\ \text{and}\;p & ={-\sigma_{zz}|}_{z=0}=-(\lambda +2\mu ){\frac{\partial w}{\partial z}|}_{z=0}-\lambda \left({\frac{\partial u}{\partial x}|}_{z=0}+{\frac{\partial v}{\partial y}|}_{z=0}\right), \end{array}\}$$

where *λ* and *μ* are Lamé's constants of an isotropic elastic material. It is useful to specify some subset of the boundary *R*, within which some given boundary values are finite and outside of which they are zero. This stems from the multitude of applications of this theory to contact problems, within which surface tractions and/or displacements are employed to model a region of contact between two bodies. In general, *R* can be any subset of the boundary plane. It may even represent a union of connected or disjoint subregions. We take (*x*′, *y*′) to be the values of the in-plane Cartesian coordinates within the region *R*. The new closed-form solutions found in this work (see appendix A) will deal specifically with general rectangular regions on the surface of the half-space, which is of the form *R* = {(*x*′, *y*′)|*a*_2_≤*x*′≤*a*_1_, *b*_2_≤*y*′≤*b*_1_}, as depicted in figure 1.

The principle of superposition simplifies a solution procedure of the combined traction boundary conditions of equation (2.1). A set of solutions specific to each of the three traction fields can be obtained separately. Each of the three sets of solutions can then be summed as a solution to the combined traction boundary value problem. The solution for non-zero normal traction is detailed in [16]. Here we provide the details of the solution of the case where *q*_*x*_ ≠ 0 and *q*_*y*_ = *p* = 0.

The equilibrium solutions of a homogeneous, isotropic, linear-elastic half-space when the traction *q*_*x*_(*x*′, *y*′) acts in the *x*-direction over the region *R* of the planar boundary can be given in terms of three potential functions

2.2 $$\psi_{x}=\int \int_{R}q_{x}(x^{\prime },y^{\prime })[z\log(z+r)-r]\;\text{d}x^{\prime }\;\text{d}y^{\prime },$$

2.3 $$\chi_{x}=\int \int_{R}q_{x}(x^{\prime },y^{\prime })\log(z+r)\;\text{d}x^{\prime }\;\text{d}y^{\prime }$$

2.4 $$\text{and}\;\;V_{x}=\int \int_{R}\frac{q_{x}(x^{\prime },y^{\prime })}{r}\;\text{d}x^{\prime }\;\text{d}y^{\prime }.$$

Here, $r=\sqrt{(x^{\prime }-x{)}^{2}+(y^{\prime }-y{)}^{2}+z^{2}}$ gives the distance between an arbitrary point (*x*′, *y*′) ∈ *R* and a point (*x*, *y*, *z*) within the half-space. These three functions satisfy the Laplace equation at each point on the half-space, i.e. Δ*ψ*_*x*_ = Δ*χ*_*x*_ = Δ*V*_*x*_ = 0; this holds for all partial derivatives of the functions. The three functions (2.2)–(2.4) are not independent, as we see that *χ*_*x*_ = ∂*ψ*_*x*_/∂*z*, and that *V*_*x*_ = ∂*χ*_*x*_/∂*z* = ∂^2^*ψ*_*x*_/∂*z*^2^. These facts become useful when calculating the closed-form solutions for specific cases. The displacements satisfying the equilibrium equations and boundary conditions are given as

2.5 $$\begin{array}{rl} u & =\frac{1}{4\pi \mu }\left[2V_{x}+\frac{\lambda }{\left(\lambda +\mu \right)}\frac{\partial^{2}\psi_{x}}{\partial x^{2}}-z\frac{\partial^{2}\chi_{x}}{\partial x^{2}}\right],\\ v & =\frac{1}{4\pi \mu }\left[\frac{\lambda }{\left(\lambda +\mu \right)}\frac{\partial^{2}\psi_{x}}{\partial x\partial y}-z\frac{\partial^{2}\chi_{x}}{\partial x\partial y}\right]\\ \text{and}\;\;w & =\frac{1}{4\pi \mu }\left[\frac{\mu }{\left(\lambda +\mu \right)}\frac{\partial \chi_{x}}{\partial x}-z\frac{\partial V_{x}}{\partial x}\right]. \end{array}\}$$

The strains are obtained from these displacements via differentiation as follows:

2.6 $$\begin{array}{rl} \varepsilon_{xx} & =\frac{\partial u}{\partial x}=\frac{1}{4\pi \mu }\left[2\frac{\partial V_{x}}{\partial x}+\frac{\lambda }{\left(\lambda +\mu \right)}\frac{\partial^{3}\psi_{x}}{\partial x^{3}}-z\frac{\partial^{3}\chi_{x}}{\partial x^{3}}\right],\\ \varepsilon_{yy} & =\frac{\partial v}{\partial y}=\frac{1}{4\pi \mu }\left[\frac{\lambda }{\left(\lambda +\mu \right)}\frac{\partial^{3}\psi_{x}}{\partial x\partial y^{2}}-z\frac{\partial^{3}\chi_{x}}{\partial x\partial y^{2}}\right],\\ \varepsilon_{zz} & =\frac{\partial w}{\partial z}=-\frac{1}{4\pi \mu }\left[\frac{\lambda }{\left(\lambda +\mu \right)}\frac{\partial V_{x}}{\partial x}+z\frac{\partial^{2}V_{x}}{\partial x\partial z}\right],\\ \varepsilon_{xy} & =\frac{1}{2}\left(\frac{\partial u}{\partial y}+\frac{\partial v}{\partial x}\right)=\frac{1}{4\pi \mu }\left[\frac{\partial V_{x}}{\partial y}+\frac{\lambda }{\left(\lambda +\mu \right)}\frac{\partial^{3}\psi_{x}}{\partial x^{2}\partial y}-z\frac{\partial^{3}\chi_{x}}{\partial x^{2}\partial y}\right],\\ \varepsilon_{xz} & =\frac{1}{2}\left(\frac{\partial u}{\partial z}+\frac{\partial w}{\partial x}\right)=\frac{1}{4\pi \mu }\left[\frac{\partial V_{x}}{\partial z}-z\frac{\partial^{2}V_{x}}{\partial x^{2}}\right]\\ \text{and}\;\varepsilon_{yz} & =\frac{1}{2}\left(\frac{\partial v}{\partial z}+\frac{\partial w}{\partial y}\right)=-\frac{z}{4\pi \mu }\left[\frac{\partial^{2}V_{x}}{\partial x\partial y}\right]. \end{array}\}$$

The stresses are obtained via Hooke's Law, along with the fact that the potentials and their derivatives all satisfy the Laplace equation. These are written in their final form as

2.7 $$\begin{array}{rl} \sigma_{xx} & =\frac{1}{2\pi }\left[\frac{3\lambda +2\mu }{\left(\lambda +\mu \right)}\frac{\partial V_{x}}{\partial x}+\frac{\lambda }{\left(\lambda +\mu \right)}\frac{\partial^{3}\psi_{x}}{\partial x^{3}}-z\frac{\partial^{3}\chi_{x}}{\partial x^{3}}\right],\\ \sigma_{yy} & =\frac{1}{2\pi }\left[\frac{\lambda }{\left(\lambda +\mu \right)}\left(\frac{\partial^{3}\psi_{x}}{\partial x\partial y^{2}}+\frac{\partial V_{x}}{\partial x}\right)-z\frac{\partial^{3}\chi_{x}}{\partial x\partial y^{2}}\right],\\ \sigma_{zz} & =-\frac{z}{2\pi }\left[\frac{\partial^{2}V_{x}}{\partial x\partial z}\right],\\ \sigma_{xy} & =\frac{1}{2\pi }\left[\frac{\partial V_{x}}{\partial y}+\frac{\lambda }{\left(\lambda +\mu \right)}\frac{\partial^{3}\psi_{x}}{\partial x^{2}\partial y}-z\frac{\partial^{3}\chi_{x}}{\partial x^{2}\partial y}\right],\\ \sigma_{xz} & =\frac{1}{2\pi }\left[\frac{\partial V_{x}}{\partial z}-z\frac{\partial^{2}V_{x}}{\partial x^{2}}\right]\\ \text{and}\;\;\sigma_{yz} & =-\frac{z}{2\pi }\left[\frac{\partial^{2}V_{x}}{\partial x\partial y}\right]. \end{array}\}$$

The boundary conditions are satisfied; *σ*_*zz*_|_*z*=0_ = *σ*_*yz*_|_*z*=0_ = 0 as assumed, and

$$\sigma_{xz}{|}_{z=0}=\frac{1}{2\pi }{\frac{\partial V_{x}}{\partial z}|}_{z=0}=\left\{ \begin{array}{cc} -q_{x}(x^{\prime },\;y^{\prime }) & \text{for}\left(x^{\prime },\;y^{\prime }\right)\in R\\ 0 & \text{for}\left(x^{\prime },\;y^{\prime }\right)\notin R \end{array}\right..$$

In practice, the problem which remains is calculating the closed forms of equations (2.2)–(2.4) and their required derivatives given the distribution of traction *q*_*x*_. A new set of calculations would need to be calculated for each given case, and the calculations involved are at best tedious and at worst impossible to obtain in the closed form. The principle of superposition, along with an interpolation scheme, offers an attractive and general alternative for addressing the problem for arbitrary tractions.

### 2.2. Solution by superposition and example calculations

Solutions for high-order contact traction boundary conditions may be obtained from the superposition of discretized solutions corresponding to lower-order interpolation functions. A tangential traction field *q*_*x*_(*x*′, *y*′) of arbitrary complexity can be approximated over a discretized loaded domain using the piecewise interpolation of bilinear polynomials. Subsequently, the lower-order solutions for each discretized subdomain are superimposed to approximate the displacement and stress fields in the elastic body. The discretization scheme is detailed in the authors' previous work [16], and thus, is omitted in the present paper. The procedure of the present solution is analogous to the collocation techniques used in the indirect boundary element method [30,31]. However, the present method incorporates the analytic calculation of the boundary integrals and superimposes the resulting functions over the whole domain, replacing a point-wise numerical quadrature scheme which would be computationally more expensive. The proposed solution method is analogous to the analytic element method, originally proposed by O.D.L. Strack to study groundwater flow [32,33]. Under this interpretation, the closed-form solutions presented here can be viewed as new ‘analytic elements’ derived for the solution of Neumann boundary value problems in an elastic half-space.

In the present work, the solution for an arbitrary distribution of tangential traction is approximated as a linear combination of potentials for constant, linear and bilinear tractions. If we divide the loaded area *R* into *M* and *N* uniform intervals in the directions of *x*′ and *y*′, respectively, then the discretization creates *M* × *N* disjoint rectangular subdomains: *R*_*ij*_ = {(*x*′, *y*′)|*x*_*i*_≤*x*′≤*x*_*i*+1_, *y*_*j*_≤*y*′≤*y*_*j*+1_} for *i* = 1, 2, …, *M* and *j* = 1, 2, …, *N* such that $R=\overset{M,N}{\underset{i,j}{⋃}}R_{ij}$. We define expressions for the potentials under the simplest polynomial traction laws over subdomains *R*_*ij*_ as

2.8 $${\text{A}}_{ij}^{mn}=\int \int_{R_{ij}}\frac{{\left(x^{\prime }\right)}^{m}{\left(y^{\prime }\right)}^{n}}{r}\;\text{d}x^{\prime }\;\text{d}y^{\prime },$$

2.9 $${\text{B}}_{ij}^{mn}=\int \int_{R_{ij}}{\left(x^{\prime }\right)}^{m}{\left(y^{\prime }\right)}^{n}\log\left(z+r\right)\;\text{d}x^{\prime }\;\text{d}y^{\prime }$$

2.10 $$\text{and}\;\;{\Gamma \;}_{ij}^{mn}=\int \int_{R_{ij}}{\left(x^{\prime }\right)}^{m}{\left(y^{\prime }\right)}^{n}[z\log(z+r)-r]\;\text{d}x^{\prime }\;\text{d}y^{\prime },$$

such that A^00^_*ij*_ is the potential of equation (2.4) when *q*_*x*_ = 1, *Γ* ^01^_*ij*_ is that of equation (2.2) when *q*_*x*_ = *y*′, etc. The present authors have calculated closed-form solutions of these functions and all derivatives present in equations (2.5)–(2.7) for constant, linear and bilinear loads, i.e. for cases *m*, *n* = 0, 1. These are listed in appendix A.

The potential defined by equation (2.2), which Boussinesq called the ‘second logarithmic potential function’, does not appear in the solution of the problem when only normal traction components have non-zero boundary values. The closed-form solutions for the corresponding equation (2.10) were therefore omitted from [16]. However, the second logarithmic potential function is one of the major difficulties in obtaining a closed-form solution to the tangential traction problem. We therefore provide some example calculations which focus on this expression.

The function ∂^3^*ψ*_*x*_/∂*x*^3^ required for the solution of the stress component *σ*_*xx*_ is approximated as

$$\frac{\partial^{3}\psi_{x}}{\partial x^{3}}\approx \sum_{i=1}^{M}\sum_{\;j=1}^{N}\left[c_{ij}^{00}\frac{\partial^{3}{\Gamma \;}_{ij}^{00}}{\partial x^{3}}+c_{ij}^{10}\frac{\partial^{3}{\Gamma \;}_{ij}^{10}}{\partial x^{3}}+c_{ij}^{01}\frac{\partial^{3}{\Gamma \;}_{ij}^{01}}{\partial x^{3}}+c_{ij}^{11}\frac{\partial^{3}{\Gamma \;}_{ij}^{11}}{\partial x^{3}}\right],$$

where *c*^*mn*^_*ij*_ are interpolation constants corresponding to the order of the polynomial components of the boundary tractions (*x*′)^*m*^(*y*′)^*n*^. For example, the last term in the bracket on the right-hand side is defined as

2.11 $$\frac{\partial^{3}{\Gamma \;}_{ij}^{11}}{\partial x^{3}}=\frac{\partial^{3}}{\partial x^{3}}\int_{y_{\;j}}^{y_{\;j+1}}\int_{x_{i}}^{x_{i+1}}x^{\prime }y^{\prime }\left[z\log(z+r)-r\right]\;\text{d}x^{\prime }\;\text{d}y^{\prime }.$$

Owing to the linearity of the integral, equation (2.11) can be rewritten as

$$\begin{array}{rl} \frac{\partial^{3}{\Gamma \;}_{ij}^{11}}{\partial x^{3}} & =\frac{\partial^{3}}{\partial x^{3}}\int_{y_{\;j}}^{y_{\;j+1}}\int_{x_{i}}^{x_{i+1}}(x^{\prime }-x)(y^{\prime }-y)\left[z\log(z+r)-r\right]\;\text{d}x^{\prime }\;\text{d}y^{\prime }+\cdots \\ & \;\frac{\partial^{3}}{\partial x^{3}}\left[x\int_{y_{\;j}}^{y_{\;j+1}}\int_{x_{i}}^{x_{i+1}}y^{\prime }\left[z\log(z+r)-r\right]\;\text{d}x^{\prime }\;\text{d}y^{\prime }\right]+\cdots \\ & \;+\frac{\partial^{3}}{\partial x^{3}}\left[y\int_{y_{\;j}}^{y_{\;j+1}}\int_{x_{i}}^{x_{i+1}}x^{\prime }\left[z\log(z+r)-r\right]\;\text{d}x^{\prime }\;\text{d}y^{\prime }\right]+\cdots \\ & \;-\frac{\partial^{3}}{\partial x^{3}}\left[xy\int_{y_{\;j}}^{y_{\;j+1}}\int_{x_{i}}^{x_{i+1}}\left[z\log(z+r)-r\right]\;\text{d}x^{\prime }\;\text{d}y^{\prime }\right]\\ & =\frac{\partial^{3}}{\partial x^{3}}\int_{y_{\;j}}^{y_{\;j+1}}\int_{x_{i}}^{x_{i+1}}(x^{\prime }-x)(y^{\prime }-y)\left[z\log(z+r)-r\right]\;\text{d}x^{\prime }\;\text{d}y^{\prime }+\cdots \\ & \;\frac{\partial^{3}}{\partial x^{3}}\left[x{\Gamma \;}_{ij}^{01}\right]+\frac{\partial^{3}}{\partial x^{3}}\left[y{\Gamma \;}_{ij}^{10}\right]-\frac{\partial^{3}}{\partial x^{3}}\left[xy{\Gamma \;}_{ij}^{00}\right]\\ & =\frac{\partial^{3}}{\partial x^{3}}\int_{y_{\;j}}^{y_{\;j+1}}\int_{x_{i}}^{x_{i+1}}(x^{\prime }-x)(y^{\prime }-y)\left[z\log(z+r)-r\right]\;\text{d}x^{\prime }\;\text{d}y^{\prime }+\cdots \\ & \;+x\frac{\partial^{3}{\Gamma \;}_{ij}^{01}}{\partial x^{3}}+3\frac{\partial^{2}{\Gamma \;}_{ij}^{01}}{\partial x^{2}}+y\frac{\partial^{3}{\Gamma \;}_{ij}^{10}}{\partial x^{3}}+\cdots \\ & \;-xy\frac{\partial^{3}{\Gamma \;}_{ij}^{11}}{\partial x^{3}}-3y\frac{\partial^{2}{\Gamma \;}_{ij}^{11}}{\partial x^{2}} \end{array}$$

The open-form integro-differential expression on the right-hand side can be found to be

$${{[\left(x^{\prime }-x\right)\left(\frac{4{\left(x^{\prime }-x\right)}^{2}+3\left({\left(y^{\prime }-y\right)}^{2}+z\left(z+r\right)\right)}{z+r}-3z\log\left(z+r\right)\right)]|}_{x_{i}}^{x_{1+1}}|}_{y_{\;j}}^{y_{\;j+1}}$$

through direct calculation by means of substitutions and reversing the orders of integrations and differentiations. This way, the function of equation (2.11) can be written in its precise closed form and evaluated over the half-space using any mathematical software package. The remaining terms on the right-hand side of equation (2.11) can be calculated in a similar manner (see appendix A).

As a second example, we consider another potential that contains partial derivatives taken with respect to both *x* and *y*:

2.12 $$\frac{\partial^{3}{\Gamma \;}_{ij}^{11}}{\partial x\partial y^{2}}=\frac{\partial^{3}}{\partial x\partial y^{2}}\int_{y_{\;j}}^{y_{\;j+1}}\int_{x_{i}}^{x_{i+1}}x^{\prime }y^{\prime }\left[z\log(z+r)-r\right]\;\text{d}x^{\prime }\;\text{d}y^{\prime }.$$

The closed-form solution of equation (2.12), which is required for the calculation of *σ*_*yy*_, can be obtained in a straightforward manner. The two differentiations with respect to *x* and *y*, cancel out the integrations with respect to *x*′ and *y*′, as per the calculation procedure of the first example:

$$\begin{array}{rl} & \frac{\partial^{3}{\Gamma \;}_{ij}^{11}}{\partial x\partial y^{2}}={{[\left(x^{\prime }-x\right)\left(\frac{{\left(y^{\prime }-y\right)}^{2}}{z+r}-\left(z\log\left(z+r\right)-r\right)\right)]|}_{x_{i}}^{x_{i+1}}|}_{y_{\;j}}^{y_{\;j+1}}+\cdots \\ & x\frac{\partial^{3}{\Gamma \;}^{01}}{\partial x\partial y^{2}}+\frac{\partial^{2}{\Gamma \;}^{01}}{\partial y^{2}}+y\frac{\partial^{3}{\Gamma \;}^{10}}{\partial x\partial y^{2}}+2\frac{\partial^{2}{\Gamma \;}^{10}}{\partial x\partial y}-xy\frac{\partial^{3}{\Gamma \;}^{00}}{\partial x\partial y^{2}}-2x\frac{\partial^{2}{\Gamma \;}^{00}}{\partial x\partial y}-y\frac{\partial^{2}{\Gamma \;}^{00}}{\partial y^{2}}-2\frac{\partial {\Gamma \;}^{00}}{\partial y}. \end{array}$$

Using the property of the harmonic function ∂*Γ*_*ij*^11^_/∂*x* (i.e. it satisfies the Laplace equation Δ(∂*Γ*_*ij*^11^_/∂*x*) = (∂^2^/∂*x*^2^ + ∂^2^/∂*y*^2^ + ∂^2^/∂*z*^2^)(∂*Γ*_*ij*^11^_/∂*x*) = 0 everywhere in the half-space), we can deduce:

2.13 $$\frac{\partial^{3}{\Gamma \;}_{ij}^{11}}{\partial x\partial z^{2}}\equiv \frac{\partial {\text{A}}_{ij}^{11}}{\partial x}=-\frac{\partial^{3}{\Gamma \;}_{ij}^{11}}{\partial x^{3}}-\frac{\partial^{3}{\Gamma \;}_{ij}^{11}}{\partial x\partial y^{2}},$$

which is required for the calculation of *σ*_*xx*_, *σ*_*yy*_ and vertical displacement *w* under tangential surface tractions acting in the *x*-direction. Similar techniques were employed to derive a complete set of closed-form solutions for the hyperbolic-paraboloidal (bilinear) tangential tractions that are listed in appendix A. Note that the variables associated with the *x*- and *y*-directions are interchangeable. Thus, the solution for the case where *q*_*y*_ ≠ 0 and *q*_*x*_ = *p* = 0 can be easily obtained. For the solution of the problem corresponding to the normal loading case, that is where *p* ≠ 0 and *q*_*x*_ = *q*_*y*_ = 0, along with the complete details of the computational procedure applied here, refer to [16].

## 3. Solution to a Cattaneo–Mindlin boundary value problem

For the validation of the solution procedure, we model two elastic spherical bodies in contact subjected to relative tangential displacements within a Hertzian normal contact interface, and benchmark on an analytical solution given by Cattaneo [34] and Mindlin [26]. A circular region of contact of radius *a* is formed between two elastically similar spheres under normal force *P*, and a subsequent tangential force *Q* is applied in the *x*-direction relative to the plane of contact. No relative displacement occurs in a ‘stick’ zone of radius *a*_stick_ < *a*, while slip occurs in the outer annulus. The resulting tangential traction *q*_*x*_ is assumed to be governed by the Coulomb model of static friction. Given the normal traction *p*, *q*_*x*_≤*c*_*f*_*p* within the extent of the contact region, where *c*_*f*_ is the coefficient of friction. The tangential contact traction *q*_*x*_ can be written as

3.1 $$q_{x}(\rho )=\left\{ \begin{array}{rl} & \frac{3c_{f}P}{2\pi a^{2}}{\left(1-\frac{\rho^{2}}{a^{2}}\right)}^{1/2}\;\;\;\;\;\;\;\;\;\;\;\;\text{for}\;a_{\mathrm{stick}}<\rho \leq a\\ & \frac{3c_{f}P}{2\pi a^{2}}{\left(1-\frac{\rho^{2}}{a^{2}}\right)}^{1/2}-\frac{3c_{\;f}Pa_{\mathrm{stick}}}{2\pi a^{3}}{\left(1-\frac{\rho^{2}}{{a_{\mathrm{stick}}}^{2}}\right)}^{1/2}\;\text{for}\;\rho \leq a_{\mathrm{stick}}, \end{array}\right.$$

where $\rho =\sqrt{x^{\prime 2}+y^{\prime 2}}$ is a radial polar coordinate within the circular contact region. Mindlin [26] provides an analytical solution of uniform tangential displacement within the stick region *ρ*≤*a*_stick_:

3.2 $$u_{\mathrm{stick}}=\frac{3c_{f}P(2-\nu )}{16\mu a}\left(1-\frac{a_{\mathrm{stick}}^{2}}{a^{2}}\right),$$

where *ν* = *λ*/2(*λ* + *μ*) is Poisson's ratio of the material. Thus, applying the tractions (3.1) in the tangential *x*-direction as a Neumann boundary condition to a homogeneous, isotropic, elastic half-space should yield the constant *x*-directional displacements of the stick region given by (3.2).

**Figure 2.**
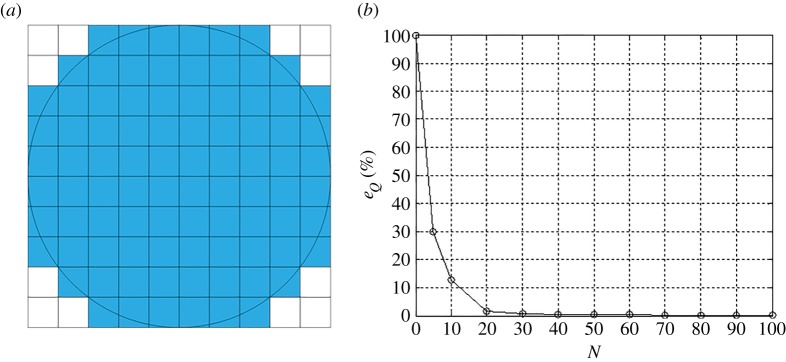
(*a*) A schematic of the discretization of a circular contact area by square subregions: the circular contact area is inscribed within a square area which is uniformly discretized by *N* × *N* square subregions. Only subdomains which overlap the circular region (shaded) contribute to the calculation; (*b*) Convergence: the sum of the integrals of the bilinear interpolants over each subdomain converges to the exact value *Q* obtained from equation (3.1) as *N* increases.

The elastic parameters used in the verification of convergence are listed in table 1. The convergence of the approximation of equation (3.1) by piecewise-bilinear surfaces is estimated in terms of the corresponding total applied tangential force. The boundary of the Hertzian circular contact region is approximated by discretization with subdomains dividing the circumscribed square, as schematically illustrated in figure 2*a*. The error in the approximation of the tangential traction field is calculated as

3.3 $$e_{Q}=\left|\frac{Q-Q_{\mathrm{approx}}}{Q}\right|\times 100\text{\%}.$$

**Figure 3.**
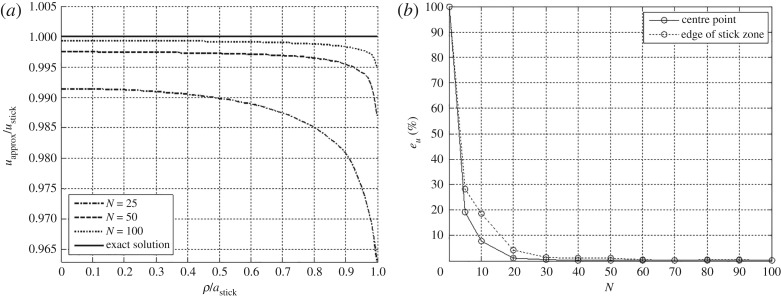
(*a*) Convergence to the predicted uniform displacement field within the stick region for the Cattaneo–Mindlin example. (*b*) Per cent error *e*_*u*_ of tangential displacement *u*_stick_ at the centre and edge of the stick region of contact from potentials superimposed for the increasing discretization of *N* × *N* square subregions approximating the circular contact area.

Figure 3 shows the convergence of the displacement ratio within the stick zone using an error estimate

3.4 $$e_{u}=\left|\frac{u_{\mathrm{stick}}-u_{\mathrm{approx}}}{u_{\mathrm{stick}}}\right|\times 100\text{\%}.$$

Both stress and displacement fields simultaneously satisfy equilibrium and compatibility. The approximation improves as a finer discretization is used to converge toward the exact circular boundary of the loaded domain using the rectilinear subdomains. However, the present discretization approximation scheme would yield a faster convergence rate when the geometry of loaded domain is rectilinear.

**Table 1.** Model parameters for approximate solutions to the Cattaneo–Mindlin problem.

normal force *P*	tangential force *Q*	*c*_*f*_	*a*	*a*_stick_
1 kgf	0.1029 kgf	0.343	1 cm	0.8879 cm

## 4. Application to settlement analysis of shallow foundations

Having verified the solution method, we undertake an application example of foundation engineering. When contact forces develop in the plane of non-conformal contact surfaces between a rigid footing and a supporting granular medium, a highly variable traction field forms at the contact plane. The corresponding equilibrium state of the supporting granular medium, and ultimately the foundation settlement, are dependent upon the boundary conditions that span multiple scales. These include, but are not limited to, variables at both the soil–structure system scale, for example, geometry and loading history [10], and the individual grain constituent scale, for example, the rheology of a rough-surface contact. In principle, the effects of intergranular forces can be quantified through statistical analysis using discrete element contact models [35–37]. However, the computational analysis required to conduct a full parametric study is daunting and costly.

An alternative for practical engineering analysis is to develop a mathematical model in which these multitudes of particulate interaction phenomena are deliberately reduced to a single quasi-static boundary value problem referring only to phenomenological observations at the foundation (system) scale. Based on a review of experimental [8–15] and analytical studies [27,28,38,39], the authors note a striking resemblance between the stress fields beneath shallow foundations and those of body force distributions in static sandpiles. The system-scale phenomenology of sandpile stresses may be best described by the FPA model [27,28]. In the FPA model, self-equilibrating tangential tractions corresponds to a unique body-force distribution that produces non-uniform normal contact pressures in the plane of contact with a rigid base plate. Initial stress equilibrium states of the shallow foundation system are then conjectured so that (i) the very existence of internal friction causes the development of self-equilibrating tangential tractions beneath vertically loaded shallow foundations and (ii) these tangential traction fields develop as per the loading history leading to a ‘saddle-shaped’ distribution of normal contact pressures, in qualitatively similar ways to the stress states observed in the sandpile experiments. The notion of coupled normal and tangential traction boundary conditions of the foundation is borne of the sandpile analogy. The working hypothesis is that tangential tractions can be empirically correlated to measured normal contact pressures, for example, measured in [12]. Thus, self-equilibrating tangential traction components can be introduced as a secondary Neumann boundary condition of the vertically loaded shallow foundation problem.

In an earlier work [16], the authors suggested an empirical function prescribing the normal-traction boundary condition for the foundation problem, which was given as

4.1 $$\;p(x^{\prime },y^{\prime })=A(x^{\prime },y^{\prime })\cos{\left(\frac{\pi }{2}\frac{\sqrt{x{{\;}^{\prime }}^{2}+y{{\;}^{\prime }}^{2}}}{\alpha (x^{\prime },y^{\prime })}\right)}^{\omega (x,y)}-B(x^{\prime },y^{\prime })\exp\left(-{\left(\frac{\sqrt{x{{\;}^{\prime }}^{2}+y{{\;}^{\prime }}^{2}}}{\zeta (x^{\prime },y^{\prime })\alpha (x^{\prime },y^{\prime })}\right)}^{2}\right).$$

**Figure 4.**
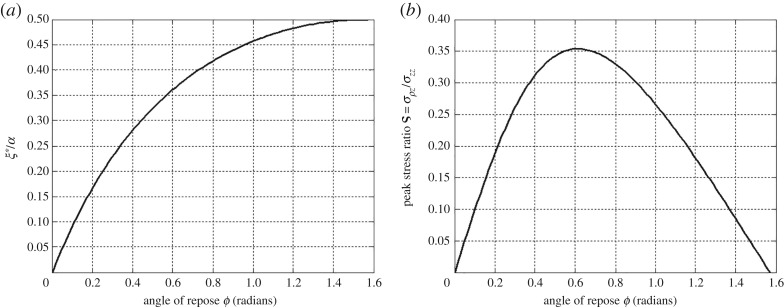
The dependence of (*a*) the location of peak pressure ξ* and (*b*) the ratio ς = *σ*_*ρz*_/*σ*_*zz*_ at the peak location on the angle of repose *ϕ* in the two-dimensional FPA model [27,28].

Here, *A*, *B*, *ζ* and *ω* form a set of auxiliary functions which vary continuously between curve-fit parameters for the point-wise contact pressures measured by Murzenko [12]; the function *α* serves effectively as mapping from a circular to a square region. A semi-empirical expression of tangential tractions can be formulated in a similar manner. We have already discussed the analogy between the normal pressures beneath shallow foundations and those found in static sandpiles, epitomized by the analytical FPA model [27,28]. The lack of empirical data of tangential contact tractions beneath foundations necessitates assumptions on these self-equilibrating tangential contact tractions in the contact plane of the vertically loaded shallow foundation. It is assumed that the location of maximum tangential traction coincides with the occurrence of the peak normal pressure. In turn, the radial location ξ* of the peak normal pressure varies with respect to the angle of repose *ϕ* where the ratio of the shear and normal stresses ς = *σ*_*ρz*_/*σ*_*zz*_ can be calculated, as shown in figure 4. In addition, the tangential traction distributions have zero values at both the centre and the edge of the contact plane. In the following, we will make use of these hypotheses to prescribe a tangential contact traction field in relation to a given normal pressure distribution measured by Murzenko [12]. It is important to note that the present numerical example is intended only to illustrate the application of the present method of solution to foundation engineering problems where traction boundary conditions are assumed to be known *a priori*.

### 4.1. Definition of tangential traction boundary conditions

Similar to equation (4.1) for normal contact traction fields, a spatial function is selected to describe the hypothesized characteristics of the tangential traction distribution across the centre and diagonal lines of a contact interface between a vertically loaded rigid footing and supporting dense granular soil. Accordingly, a single-variable function can be defined as

**Figure 5.**
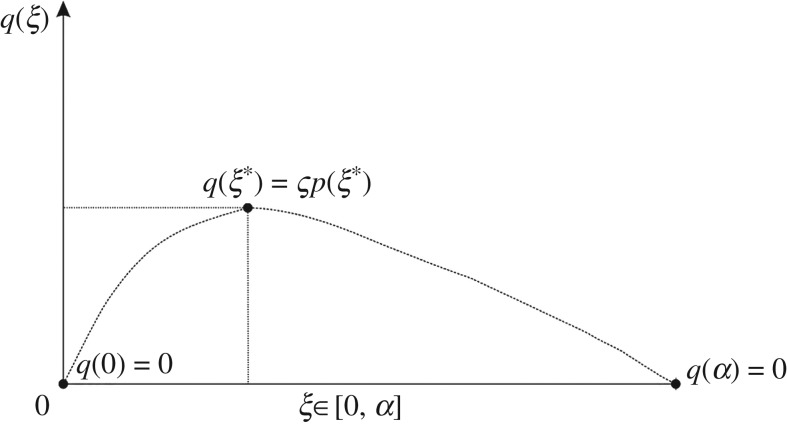
A schematic outline of a parameter-fitting procedure performed with equation (4.2) for cross-sectional shear tractions across the centre line and diagonal of the square region *R*_*S*_.

4.2 $$q(\xi )=C\xi (\alpha -\xi )\;{\text{e}}^{(\left(\alpha -\xi \right)/D{)}^{2}},$$

where *C* and *D* are free parameters for the *α* is the distance from the centroid to the edge of the loaded region. *ξ* is a local coordinate defined along the lines, as outlined in figure 5. Referring to the peak location of the normal traction ξ* , the free parameters of equation (4.2) are subjected to two conditions.

**Figure 6.**
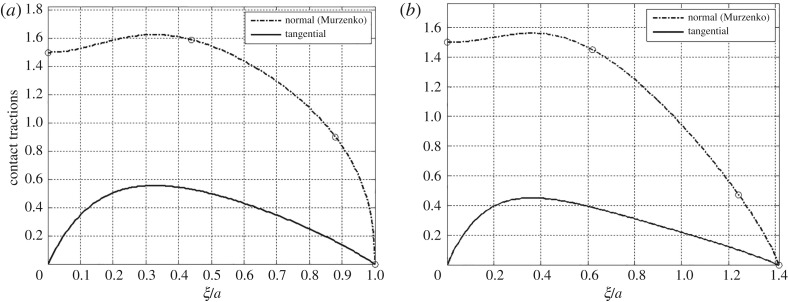
The comparison of normal and shear tractions (*a*) across the centre line and (*b*) across the diagonal line of the loaded domain from equations (4.2) and (4.1), respectively. The normal traction curves are curve-fit from Murzenko [12], (cycle IX, average pressure 1 kgf cm^−2^). The necessary parameters for these cases are given in table 2.

Stated mathematically, the first condition is d*q*/d*ξ*|_*ξ*=ξ*_ = 0. The second condition is that the peak magnitude of the tangential traction, *q*(ξ*), is set equal to ς*p*(ξ*), where ς is the proportionality at the peak location. It is physically plausible to let ς be the stress ratio *σ*_*ρz*_/*σ*_*zz*_ of the FPA model. We obtain the peak locations across the centre and diagonal lines for the normal contact pressure data of Murzenko [12] as per equation (4.1) with the parameters given in table 2. These peak locations are then correlated with angles of repose from the FPA model using figure 4*a*. The angles of repose in the present example are intended as an intermediary curve-fit parameter of the working hypothesis, used to correlate point-wise measurements of normal contact pressure with statically admissible stress states of granular materials from the FPA model. They are not meant to be interpreted as physical material properties of Murzenko's sand. From figure 4*b*, the values of the peak stress ratio are found to be ς_0_ = 0.3429 and ς_45_ = 0.2890, where the subscripts refer to the angles across which the fitting occurs, that is the centre and diagonal lines of the loaded square region. In figure 6, the tangential tractions are shown for comparison with the curve-fitted normal contact pressure distributions. All relevant parameters for the generation of these expressions from equations (4.1) and (4.2) are given in table 2.

**Table 2.** Selected parameters chosen for equations (4.1) and (4.2) for shear and normal tractions, respectively. The subscripts 0 and 45 refer to the tractions across the centre line and diagonal of the square loaded region, respectively.

*A*_0_	*A*_45_	*B*_0_	*B*_45_	*ω*_0_	*ω*_45_
1.7939	1.8292	0.2939	0.3292	0.4119	0.8114

*ζ*_0_	*ζ*_45_	*C*_0_	*C*_45_	*D*_0_	*D*_45_
0.2715	0.2853	5.9460	1.8308	0.4609	0.5390

Next, let *ρ* and *θ* be a set of polar coordinates over the loaded square region. Using the same procedure as outlined in [16], one can obtain a continuous expression of tangential radial traction *q*_*ρ*_(*ρ*, *θ*):

4.3 $$q_{\rho }(\rho ,\theta )=C(\theta )\rho (\alpha (\theta )-\rho )e^{{\left(\left(\alpha (\theta )-\rho \right)/D(\theta )\right)}^{2}}.$$

Here, the auxiliary functions are defined as

$$\begin{array}{rl} & C(\theta )=C_{0}f\;{\left(\theta \right)}^{2\log(C_{45}/C_{0})/\log(2),}\\ & D(\theta )=D_{0}f\;{\left(\theta \right)}^{2\log(D_{45}/D_{0})/\log(2)}\\ \text{and}\;\; & \alpha (\theta )=af(\theta ), \end{array}$$

where log stands for the natural logarithm. The function f(θ)=min[1/|cos⁡(θ)|, 1/|sin⁡(θ)|] maps the parameters to the square region in the desired fashion.

Finally, the Cartesian components of the radial traction *q*_*ρ*_(*ρ*, *θ*) can be defined so that we may prescribe the desired boundary conditions in our coordinate system of choice. Transforming equation (4.3) into Cartesian coordinates by substituting $\rho (x^{\prime },y^{\prime })=\sqrt{x^{\prime \;2}+y^{\prime \;2}}$ and *θ*(*x*′, *y*′) = tan^−1^(*y*′/*x*′), we obtain

4.4 $$\begin{array}{cc} & q_{x}(x^{\prime },y^{\prime })=\cos\left(\theta (x^{\prime },y^{\prime })\right)q_{\rho }(\rho (x^{\prime },y^{\prime }),\;\theta (x^{\prime },y^{\prime }))\\ \text{and}\;\; & q_{y}(x^{\prime },y^{\prime })=\sin\left(\theta (x^{\prime },y^{\prime })\right)q_{\rho }(\rho (x^{\prime },y^{\prime }),\;\theta (x^{\prime },y^{\prime })). \end{array}\}$$

**Figure 7.**
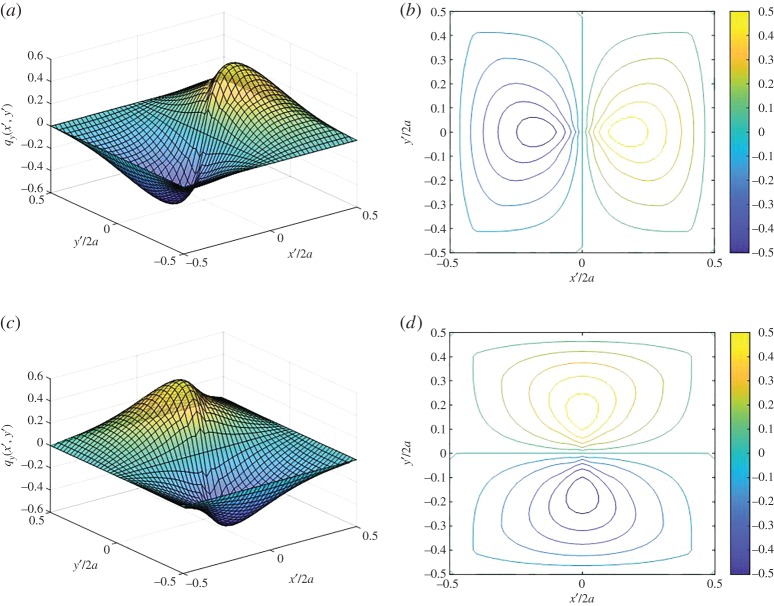
(*a*) Surface plot of traction field *q*_*x*_. (*b*) Contour of *q*_*x*_. (*c*) Surface plot of traction field *q*_*y*_. (*d*) Contour of *q*_*y*_.

Surface tractions *q*_*x*_ and *q*_*y*_, which are obtained from equations (4.3) and (4.4) for the parameters in table 2, are graphically illustrated in surface plots and contours in the Cartesian coordinate system in figure 7. Solutions to both the *x*- and *y*-directional surface traction boundary conditions can be superimposed with the solution to the normal traction boundary condition [16] to obtain a new stress equilibrium state.

### 4.2. Numerical results from combined boundary conditions

With a discretization scheme of 40 × 40 subdomains across the square domain *R*_*S*_ of the rigid footing, total *z*-directional displacement *w* at the contact boundary plane *z* = 0 is investigated in relation to the combined influence of the tangential and normal surface traction boundary conditions. These influences are defined separately as follows:

4.5 $$\begin{array}{rl} {w_{q}|}_{z=0} & ={w_{q_{x}}|}_{z=0}+{w_{q_{y}}|}_{z=0}=\frac{\left(1+\nu )(1-2\nu \right)}{2\pi E}\left({\frac{\partial \chi_{x}}{\partial x}|}_{z=0}+{\frac{\partial \chi_{y}}{\partial y}|}_{z=0}\right)\\ \text{and}\;\;\;\;{w_{\;p}|}_{z=0} & =\frac{1}{4\pi \mu }\frac{\lambda +2\mu }{\lambda +\mu }\left({V_{z}|}_{z=0}\right)=\frac{1-\nu^{2}}{E\pi }\left({V_{z}|}_{z=0}\right). \end{array}\}$$

Here, the potentials *χ* and *V* are those defined in equations (2.3) and (2.4); the subscripts (*x*, *y*, *z*) are used here to specify these potentials under the action of the Cartesian surface tractions (*q*_*x*_, *q*_*y*_, *p*). Subsequently, the total vertical displacement is written as *w*|_*z*=0_ = *w*_*q*_|_*z*=0_ + *w*_*p*_|_*z*=0_. We can define corresponding expressions for these surface displacements normalized by the elastic constants and the width of the loaded area as follows:

4.6 $$\begin{array}{rl} w_{q}^{*} & \equiv \frac{E}{2a(1+\nu )(1-2\nu )}{w_{q}|}_{z=0}=\frac{1}{4\pi a}\left({\frac{\partial \chi_{x}}{\partial x}|}_{z=0}+{\frac{\partial \chi_{y}}{\partial y}|}_{z=0}\right)\\ \text{and}\;\;\;\;w_{\;p}^{*} & \equiv \frac{E}{2a(1-\nu^{2})}{w_{\;p}|}_{z=0}=\frac{1}{2\pi a}{V_{z}|}_{z=0}. \end{array}\}$$

**Figure 8.**
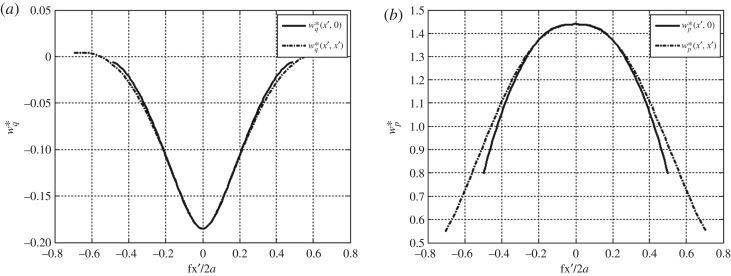
Normalized vertical surface displacements (*a*) w*_q_ induced by the combined tangential tractions and (*b*) w*_p_ from the vertical normal tractions. These are each given as cross-sectional curves across the centre and diagonal of the loaded region.

As shown in figure 8, the components of normalized vertical displacement defined in equations (4.6) are given as the cross-sections across the centre and diagonal lines of the loaded region. It is noted that the vertical displacement due to the applied tangential traction is of opposite sign to that resulting from the normal (compressive) traction. Therefore, the inclusion of the tangential tractions has the effect of increasing vertical resistances in the foundation system. For example, with elastic constants *E* = 1000 kgf cm^−2^ and *ν* = 0.4, the difference in maximum vertical displacement is about 4.3%. The peak displacement of the normal traction boundary condition only is approximately 12.09 × 10^−4^ cm, whereas that of the tangential traction boundary condition is approximately −5.15 × 10^−5^ cm for loads applied over a unit area.

**Figure 9.**
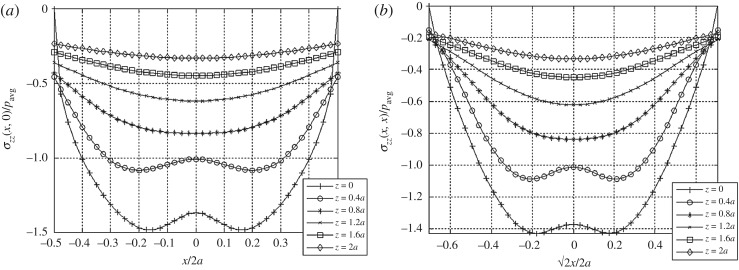
Vertical stress distributions at various depths beneath the loaded area (*a*) across the centre line and (*b*) the diagonal for the combined normal and tangential boundary tractions. The effect of the tangential tractions is to decrease the normal compressive stress beneath the load, thus increasing the range in which the distributions exhibit a pressure dip.

Although the difference is small in the case of vertical displacements, which are mainly due to normal tractions, the presence of tangential tractions has an interesting effect on the vertical stress field *σ*_*zz*_ developed in the elastic body (figure 9). In comparison with the vertical stresses obtained from the normal tractions alone [16], their distributions show more pronounced pressure dips for a larger region with respect to depth. This suggests that the tangential traction boundary condition of the linear elastic foundation system may be calibrated to account for the confining effect of geostatic stresses in the development of vertical resistances.

**Figure 10.**
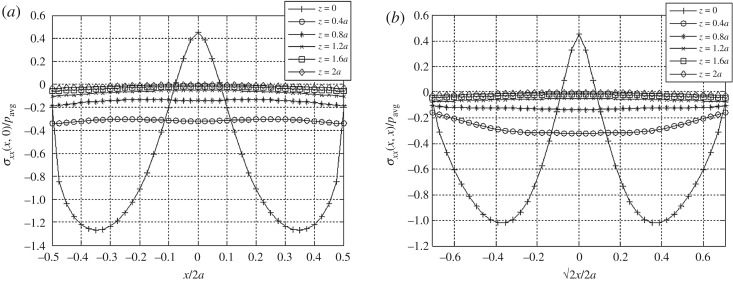
Distributions of *σ*_*xx*_ at various depths beneath the loaded area (*a*) across the centre line and (*b*) the diagonal for the combined normal and tangential boundary tractions. Calculations performed for Young's modulus *E* = 1000 kgf cm^−2^ and Poisson's ratio *ν* = 0.4.

It is noteworthy that tensile horizontal stresses *σ*_*xx*_ develop in the centre within a shallow depth beneath the contact plane (figure 10). This simulated surface tension counteracts the load effect of applied vertical force in the elastic body. In the context of soil mechanics, the pre-stressed contact surface in tension can be interpreted as the confining effects everywhere within a shallow depth beneath a rigid footing (i.e. mobilized shearing resistance of a granular material against downward vertical displacements). However, the use of tangential traction boundary conditions to mimic the observed quasi-static bulk shear behaviours of granular materials [40] is subject to further validation. A systematic phenomenology is required that correlates the self-affine nature of granular soil at the underlying scales, for example, the topological and metric properties of fractal structures [41], to the system-scale contact phenomena.

## 5. Concluding remarks

In the present study, an analysis technique is developed to solve generalized Neumann boundary value problems (i.e. a homogeneous and isotropic elastic half-space subjected to an arbitrary distribution of combined normal and tangential tractions on its surface). The method for the proposed solution uses the interpolation and superposition of the closed-form solutions of potential functions for bilinear boundary conditions over rectangular loaded domains. The motivation has been computationally efficient approximate solutions to foundation engineering contact problems. The simulations presented here predict that the hypothetical presence of unique tangential traction fields impedes vertical displacement and produces pretension zones that lead to the development of shearing resistance. In addition, these zones alter the stress states of the supporting elastic body and tend to attenuate the rise of compressive vertical stress at the centre of the foundation. It is therefore evident that tangentially induced stresses in the contact plane are related not only to externally applied tangential loading, but also to internal friction (e.g. angle of repose [27,28]) and loading history [10,12].

The model presented here offers an improved tool for studying multidimensional contact evolution inside a loaded region subjected to combined loading conditions. For example, a comprehensive experimental study of contact stresses that is beyond the scope of this paper is necessary to characterize the range of physically plausible boundary conditions for the foundation engineering problem. The authors hope that the present solution's method, which is an extension of earlier work [16], will serve as a basis for researchers to explore a wide range of other applications in contact mechanics on various scales [42].

## Supplementary Material

Analysis data for graphs

## Data accessibility

All data and source code required to generate the results presented here have been included as the electronic supplementary material.

## Appendix A. Library of closed-form potentials

The closed-form solutions required to apply our computational procedure to the solutions in this paper are listed here. We give the solutions in general for arbitrary rectangular regions *R* = {(*x*′, *y*′, 0)|*a*_2_≤*x*′≤*a*_1_, *b*_2_≤*y*′≤*b*_1_}. Note that in the following, the notation []|^*a*_1_^_*a*_2__|^*b*_1_^_*b*_2__ is used to compact the multiple repeated terms generated by the double definite integration by the fundamental theorem of calculus. In general, if *F*(*x*′, *y*′) is a function of two variables, the operator acts upon it as follows:

$$[F(x^{\prime },y^{\prime })]{|}_{a_{2}}^{a_{1}}{|}_{b_{2}}^{b_{1}}=(F(a_{1},\;b_{1})-F(a_{1},\;b_{2}))-(F(a_{2},\;b_{1})-F(a_{2},\;b_{2})t).$$

Ellipses ‘ … ’ are used to carry on to the next line when the analytic expressions become too long. For the sake of brevity, we omit a number of expressions (e.g. ∂*Γ*^00^/∂*y*, ∂^2^*Γ*^01^/∂*y*^2^), which can be obtained through symmetry from other results given (e.g. ∂*Γ*^00^/∂*x*, ∂^2^*Γ*^10^/∂*x*^2^).

**A.1. Constant traction solutions**

$$\begin{array}{rl} & {\text{A}}^{00}={{[(y^{\prime }-y)\log\left(r+x^{\prime }-x\right)+(x^{\prime }-x)\log\left(r-y+y^{\prime }\right)-z\tan^{-1}\left(\frac{\left(x^{\prime }-x)(y^{\prime }-y\right)}{zr}\right)]|}_{a_{2}}^{a_{1}}|}_{b_{2}}^{b_{1}}\\ & \frac{\partial {\text{A}}^{00}}{\partial x}={{[-\log\left(r+y^{\prime }-y\right)]|}_{a_{2}}^{a_{1}}|}_{b_{2}}^{b_{1}}\\ & \frac{\partial^{2}{\text{A}}^{00}}{\partial x^{2}}={{[-\frac{\left(x^{\prime }-x\right)\left(y^{\prime }-y\right)}{\left({\left(x^{\prime }-x\right)}^{2}+z^{2}\right)r}]|}_{a_{2}}^{a_{1}}|}_{b_{2}}^{b_{1}}\\ & \frac{\partial {\text{A}}^{00}}{\partial z}={{[-\tan^{-1}\left(\frac{\left(x^{\prime }-x\right)\left(y^{\prime }-y\right)}{zr}\right)]|}_{a_{2}}^{a_{1}}|}_{b_{2}}^{b_{1}}\\ & \frac{\partial^{2}{\text{A}}^{00}}{\partial x\partial z}={{[\frac{\left(y^{\prime }-y\right)z}{\left({\left(x^{\prime }-x\right)}^{2}+z^{2}\right)r}]|}_{a_{2}}^{a_{1}}|}_{b_{2}}^{b_{1}}\\ & \frac{\partial^{2}{\text{A}}^{00}}{\partial x\partial y}={{[\frac{1}{r}]|}_{a_{2}}^{a_{1}}|}_{b_{2}}^{b_{1}}\\ & {\text{B}}^{00}={{[\begin{array}{cc} & \;-\frac{3}{2}\left(x^{\prime }-x\right)\left(y^{\prime }-y\right)+\frac{1}{2}{\left(x^{\prime }-x\right)}^{2}\left(\tan^{-1}\left(\frac{y^{\prime }-y}{x^{\prime }-x}\right)-\tan^{-1}\left(\frac{z\left(y^{\prime }-y\right)}{\left(x^{\prime }-x\right)r}\right)\right)+\cdots \\ & \;\frac{1}{2}{\left(y^{\prime }-y\right)}^{2}\left(\tan^{-1}\left(\frac{x^{\prime }-x}{y^{\prime }-y}\right)-\tan^{-1}\left(\frac{z\left(x^{\prime }-x\right)}{\left(y^{\prime }-y\right)r}\right)\right)-\frac{1}{2}z^{2}\tan^{-1}\left(\frac{\left(x^{\prime }-x\right)\left(y^{\prime }-y\right)}{zr}\right)+\cdots \\ & z\left(x^{\prime }-x\right)\log\left(y^{\prime }-y+r\right)+z\left(y^{\prime }-y\right)\log\left(x^{\prime }-x+r\right)+\left(x^{\prime }-x\right)\left(y^{\prime }-y\right)\log\left(z+r\right) \end{array}]|}_{a_{2}}^{a_{1}}|}_{b_{2}}^{b_{1}} \end{array}$$

$$\begin{array}{rl} & \frac{\partial {\text{B}}^{00}}{\partial x}={{[\begin{array}{cc} & \left(x^{\prime }-x\right)\left(\tan^{-1}\left(\frac{\left(y^{\prime }-y\right)z}{\left(x^{\prime }-x\right)r}\right)-\tan^{-1}\left(\frac{y^{\prime }-y}{x^{\prime }-x}\right)\right)+\cdots \\ & -z\log\left(\left(y^{\prime }-y\right)+r\right)-\left(y^{\prime }-y\right)\log\left(z+r\right) \end{array}]|}_{a_{2}}^{a_{1}}|}_{b_{2}}^{b_{1}}\\ & \frac{\partial^{2}{\text{B}}^{00}}{\partial x^{2}}{={[\tan^{-1}\left(\frac{y^{\prime }-y}{x^{\prime }-x}\right)-\tan^{-1}\left(\frac{z\left(y^{\prime }-y\right)}{\left(x^{\prime }-x\right)r}\right)]|}_{a_{2}}^{a_{1}}|}_{b_{2}}^{b_{1}}\\ & \frac{\partial^{3}{\text{B}}^{00}}{\partial x^{3}}{={[\frac{y^{\prime }-y}{{\left(x^{\prime }-x\right)}^{2}+{\left(y^{\prime }-y\right)}^{2}}-\frac{\left(y^{\prime }-y\right)z\left({\left(x^{\prime }-x\right)}^{2}+r^{2}\right)}{\left({\left(x^{\prime }-x\right)}^{2}+{\left(y^{\prime }-y\right)}^{2}\right)\left({\left(x^{\prime }-x\right)}^{2}+z^{2}\right)r}]|}_{a_{2}}^{a_{1}}|}_{b_{2}}^{b_{1}}\\ & \frac{\partial^{2}{\text{B}}^{00}}{\partial x\partial y}={{[\log\left(z+r\right)]|}_{a_{2}}^{a_{1}}|}_{b_{2}}^{b_{1}}\\ & \frac{\partial^{3}{\text{B}}^{00}}{\partial x^{2}\partial y}={{[-\frac{x^{\prime }-x}{r\left(z+r\right)}]|}_{a_{2}}^{a_{1}}|}_{b_{2}}^{b_{1}} \end{array}$$

$$\begin{array}{rl} & {\Gamma \;}^{00}={{[\begin{array}{cc} & \;-\frac{3}{2}\left(x^{\prime }-x\right)\left(y^{\prime }-y\right)z+\frac{1}{2}{\left(x^{\prime }-x\right)}^{2}z\left(\tan^{-1}\left(\frac{y^{\prime }-y}{x^{\prime }-x}\right)-\tan^{-1}\left(\frac{z\left(y^{\prime }-y\right)}{\left(x^{\prime }-x\right)r}\right)\right)+\cdots \\ & \;\frac{1}{2}{\left(y^{\prime }-y\right)}^{2}z\left(\tan^{-1}\left(\frac{x^{\prime }-x}{y^{\prime }-y}\right)-\tan^{-1}\left(\frac{z\left(x^{\prime }-x\right)}{\left(y^{\prime }-y\right)r}\right)\right)-\frac{1}{6}z^{3}\tan^{-1}\left(\frac{\left(x^{\prime }-x\right)\left(y^{\prime }-y\right)}{zr}\right)+\cdots \\ & \;-\frac{1}{3}\left(x^{\prime }-x\right)\left(y^{\prime }-y\right)r-\frac{1}{6}{\left(x^{\prime }-x\right)}^{3}\log\left(y^{\prime }-y+r\right)+\frac{1}{2}z^{2}\left(x^{\prime }-x\right)\log\left(y^{\prime }-y+r\right)+\cdots \\ & -\frac{1}{6}{\left(y^{\prime }-y\right)}^{3}\log\left(x^{\prime }-x+r\right)+\frac{1}{2}z^{2}\left(y^{\prime }-y\right)\log\left(x^{\prime }-x+r\right)+\left(x^{\prime }-x\right)\left(y^{\prime }-y\right)z\log\left(z+r\right) \end{array}]|}_{a_{2}}^{a_{1}}|}_{b_{2}}^{b_{1}}\\ & \frac{\partial {\Gamma \;}^{00}}{\partial x}={{[\begin{array}{cc} & \frac{1}{2}\left(y^{\prime }-y\right)r-\left(x^{\prime }-x\right)z\left(\tan^{-1}\left(\frac{\left(y^{\prime }-y\right)}{\left(x^{\prime }-x\right)}\right)-\tan^{-1}\left(\frac{z(y^{\prime }-y)}{(x^{\prime }-x)r}\right)\right)+\cdots \\ & \;\frac{1}{2}\left({\left(x^{\prime }-x\right)}^{2}-z^{2}\right)\log\left(r+y^{\prime }-y\right)-\left(y^{\prime }-y\right)z\log\left(r+z\right) \end{array}]|}_{a_{2}}^{a_{1}}|}_{b_{2}}^{b_{1}}\\ & \frac{\partial^{2}{\Gamma \;}^{00}}{\partial x^{2}}={{[-(x^{\prime }-x)\log\left(r-y+y^{\prime }\right)+z\left(\tan^{-1}\left(\frac{\left(y^{\prime }-y\right)}{\left(x^{\prime }-x\right)}\right)-\tan^{-1}\left(\frac{z(y^{\prime }-y)}{(x^{\prime }-x)r}\right)\right)]|}_{a_{2}}^{a_{1}}|}_{b_{2}}^{b_{1}}\\ & \frac{\partial^{3}{\Gamma \;}^{00}}{\partial x^{3}}={{[\frac{\left(y^{\prime }-y\right)\left(z-r\right)}{{\left(x^{\prime }-x\right)}^{2}+{\left(y^{\prime }-y\right)}^{2}}+\log\left(y^{\prime }-y+r\right)]|}_{a_{2}}^{a_{1}}|}_{b_{2}}^{b_{1}}\\ & {\frac{\partial^{2}{\Gamma \;}^{00}}{\partial x\partial y}={[z\log\left(r+z\right)-r]|}_{a_{2}}^{a_{1}}|}_{b_{2}}^{b_{1}}\\ & \frac{\partial^{3}{\Gamma \;}^{00}}{\partial x^{2}\partial y}={{[\frac{x^{\prime }-x}{z+r}]|}_{a_{2}}^{a_{1}}|}_{b_{2}}^{b_{1}} \end{array}$$

**A.2. x-Linear traction solutions**

$$\;\begin{array}{rl} {\text{A}}^{10} & ={{[\frac{1}{2}\left(y^{\prime }-y\right)r+\frac{1}{2}\left({\left(x^{\prime }-x\right)}^{2}+z^{2}\right)\log\left(r+y^{\prime }-y\right)]|}_{a_{2}}^{a_{1}}|}_{b_{2}}^{b_{1}}+x{\text{A}}^{00}\\ \frac{\partial {\text{A}}^{10}}{\partial x} & ={{[-\left(x^{\prime }-x\right)\log\left(y^{\prime }-y+r\right)]|}_{a_{2}}^{a_{1}}|}_{b_{2}}^{b_{1}}+x\frac{\partial {\text{A}}^{00}}{\partial x}+{\text{A}}^{00}\\ \frac{\partial^{2}{\text{A}}^{10}}{\partial x^{2}} & ={{[\log\left(r+y^{\prime }-y\right)-\frac{{\left(x^{\prime }-x\right)}^{2}\left(y^{\prime }-y\right)}{\left({\left(x^{\prime }-x\right)}^{2}+z^{2}\right)r}]|}_{a_{2}}^{a_{1}}|}_{b_{2}}^{b_{1}}+x\frac{\partial^{2}{\text{A}}^{00}}{\partial x^{2}}+2\frac{\partial {\text{A}}^{00}}{\partial x}\\ \frac{\partial {\text{A}}^{10}}{\partial z} & ={{[z\log\left(r+y^{\prime }-y\right)]|}_{a_{2}}^{a_{1}}|}_{b_{2}}^{b_{1}}+x\frac{\partial {\text{A}}^{00}}{\partial z} \end{array}$$

$$\begin{array}{rl} \frac{\partial^{2}{\text{A}}^{10}}{\partial x\partial z} & ={{[\frac{\left(x^{\prime }-x\right)\left(y^{\prime }-y\right)z}{\left({\left(x^{\prime }-x\right)}^{2}+z^{2}\right)r}]|}_{a_{2}}^{a_{1}}|}_{b_{2}}^{b_{1}}+x\frac{\partial^{2}{\text{A}}^{00}}{\partial x\partial z}+\frac{\partial {\text{A}}^{00}}{\partial z}\\ \frac{\partial^{2}{\text{A}}^{10}}{\partial x\partial y} & ={{[\frac{\left(x^{\prime }-x\right)}{r}]|}_{a_{2}}^{a_{1}}|}_{b_{2}}^{b_{1}}+x\frac{\partial^{2}{\text{A}}^{00}}{\partial x\partial y}+\frac{\partial {\text{A}}^{00}}{\partial y}\\ {\text{B}}^{10} & =\frac{1}{18}{{[\begin{array}{cc} & \;6{\left(x^{\prime }-x\right)}^{3}\left(\tan^{-1}\left(\frac{\left(y^{\prime }-y\right)}{\left(x^{\prime }-x\right)}\right)-\tan^{-1}\left(\frac{\left(y^{\prime }-y\right)z}{\left(x^{\prime }-x\right)r}\right)\right)+\cdots \\ & \;3z\left(3{\left(x^{\prime }-x\right)}^{2}+z^{2}\right)\log\left(y^{\prime }-y+r\right)+9\left(y^{\prime }-y\right){\left(x^{\prime }-x\right)}^{2}\log\left(z+r\right)+\cdots \\ & \;\left(y^{\prime }-y\right)\left(-{\left(y^{\prime }-y\right)}^{2}-6{\left(x^{\prime }-x\right)}^{2}+6zr+3{\left(y^{\prime }-y\right)}^{2}\log\left(z+r\right)\right) \end{array}]|}_{a_{2}}^{a_{1}}|}_{b_{2}}^{b_{1}}+x{\text{B}}^{00} \end{array}$$

$$\begin{array}{rl} \frac{\partial {\text{B}}^{10}}{\partial x} & ={{[\begin{array}{cc} & \;{\left(x^{\prime }-x\right)}^{2}\left(\tan^{-1}\left(\frac{\left(y^{\prime }-y\right)z}{\left(x^{\prime }-x\right)r}\right)-\tan^{-1}\left(\frac{y^{\prime }-y}{x^{\prime }-x}\right)\right)+\cdots \\ & \;-z\left(x^{\prime }-x\right)\log\left(\left(y^{\prime }-y\right)+r\right)-\left(x^{\prime }-x\right)\left(y^{\prime }-y\right)\log\left(z+r\right)+\cdots \\ & (x^{\prime }-x)y^{\prime } \end{array}]|}_{a_{2}}^{a_{1}}|}_{b_{2}}^{b_{1}}+x\frac{\partial {\text{B}}^{00}}{\partial x}+{\text{B}}^{00}\\ \frac{\partial^{2}{\text{B}}^{10}}{\partial x^{2}} & ={{[\begin{array}{cc} & \;2\left(x^{\prime }-x\right)\left(\tan^{-1}\left(\frac{y^{\prime }-y}{x^{\prime }-x}\right)-\tan^{-1}\left(\frac{\left(y^{\prime }-y\right)z}{\left(x^{\prime }-x\right)r}\right)\right)+\cdots \\ & \;z\log\left(\left(y^{\prime }-y\right)+r\right)+\left(y^{\prime }-y\right)\log\left(z+r\right) \end{array}]|}_{a_{2}}^{a_{1}}|}_{b_{2}}^{b_{1}}+\;x\frac{\partial^{2}{\text{B}}^{00}}{\partial x^{2}}+2\frac{\partial {\text{B}}^{00}}{\partial x}\\ \frac{\partial {\text{B}}^{10}}{\partial x^{3}} & ={{[\begin{array}{cc} & \;2\left(\tan^{-1}\left(\frac{\left(y^{\prime }-y\right)}{\left(x^{\prime }-x\right)}\right)-\tan^{-1}\left(\frac{\left(y^{\prime }-y\right)z}{\left(x^{\prime }-x\right)r}\right)\right)+\cdots \\ & \;\frac{2\left(x^{\prime }-x\right)\left(y^{\prime }-y\right)}{{\left(x^{\prime }-x\right)}^{2}+{\left(y^{\prime }-y\right)}^{2}}-\frac{\left(x^{\prime }-x\right)z}{r\left(y^{\prime }-y+r\right)}+\cdots \\ & \;-\frac{\left(x^{\prime }-x\right)\left(y^{\prime }-y\right)}{r\left(z+r\right)}-\frac{2\left(x^{\prime }-x\right)\left(y^{\prime }-y\right)z\left(2{\left(x^{\prime }-x\right)}^{2}+{\left(y^{\prime }-y\right)}^{2}+z^{2}\right)}{\left({\left(x^{\prime }-x\right)}^{2}+{\left(y^{\prime }-y\right)}^{2}\right)\left({\left(x^{\prime }-x\right)}^{2}+z^{2}\right)r} \end{array}]|}_{a_{2}}^{a_{1}}|}_{b_{2}}^{b_{1}}+\cdots \\ & \;+x\frac{\partial^{3}{\text{B}}^{00}}{\partial x^{3}}+3\frac{\partial^{2}{\text{B}}^{00}}{\partial x^{2}} \end{array}$$

$$\begin{array}{rl} \frac{\partial^{2}{\text{B}}^{10}}{\partial x\partial y} & ={{[\left(x^{\prime }-x\right)\log\left(z+r\right)]|}_{a_{2}}^{a_{1}}|}_{b_{2}}^{b_{1}}+x\frac{\partial^{2}{\text{B}}^{00}}{\partial x\partial y}+\frac{\partial {\text{B}}^{00}}{\partial y}\\ \frac{\partial^{3}{\text{B}}^{10}}{\partial x^{2}\partial y} & ={{[-\frac{{\left(x^{\prime }-x\right)}^{2}}{r(z+r)}-\log\left(z+r\right)]|}_{a_{2}}^{a_{1}}|}_{b_{2}}^{b_{1}}+x\frac{\partial^{3}{\text{B}}^{00}}{\partial x^{2}\partial y}+2\frac{\partial^{2}{\text{B}}^{00}}{\partial x\partial y}\\ \frac{\partial {\Gamma \;}^{10}}{\partial x} & ={{[\frac{1}{2}\left(x^{\prime }-x\right)\left(\begin{array}{cc} & \;2y^{\prime }z+\left(y^{\prime }-y\right)r+\cdots \\ & \;-2\left(x^{\prime }-x\right)z\left(\tan^{-1}\left(\frac{\left(y^{\prime }-y\right)}{\left(x^{\prime }-x\right)}\right)-\tan^{-1}\left(\frac{\left(y^{\prime }-y\right)z}{\left(x^{\prime }-x\right)r}\right)\right)+\cdots \\ & \;\left({\left(x^{\prime }-x\right)}^{2}-z^{2}\right)\log\left(y^{\prime }-y+r\right)-2\left(y^{\prime }-y\right)z\log\left(z+r\right) \end{array}\right)]|}_{a_{2}}^{a_{1}}|}_{b_{2}}^{b_{1}}+\cdots \\ & \;+x\frac{\partial {\Gamma \;}^{00}}{\partial x}+{\Gamma \;}^{00} \end{array}$$

$$\begin{array}{rl} \frac{\partial^{2}{\Gamma \;}^{10}}{\partial x^{2}} & ={{[\begin{array}{cc} & -\frac{1}{2}\left(y^{\prime }-y\right)r+2\left(x^{\prime }-x\right)z\left(\tan^{-1}\left(\frac{y^{\prime }-y}{x^{\prime }-x}\right)-\tan^{-1}\left(\frac{\left(y^{\prime }-y\right)z}{\left(x^{\prime }-x\right)r}\right)\right)+\cdots \\ & -\frac{1}{2}\left(3{\left(x^{\prime }-x\right)}^{2}-z^{2}\right)\log\left(y^{\prime }-y+r\right)+\left(y^{\prime }-y\right)z\log\left(z+r\right)+ \end{array}]|}_{a_{2}}^{a_{1}}|}_{b_{2}}^{b_{1}}+\cdots \\ & \;+x\frac{\partial^{2}{\Gamma \;}^{00}}{\partial x^{2}}+2\frac{\partial {\Gamma \;}^{00}}{\partial x}\\ \frac{\partial^{3}{\Gamma \;}^{10}}{\partial x^{3}} & ={{[\begin{array}{cc} & -\frac{\left(x^{\prime }-x\right)\left(y^{\prime }-y\right)\left(r-z\right)}{{\left(x^{\prime }-x\right)}^{2}+{\left(y^{\prime }-y\right)}^{2}}-2z\left(\tan^{-1}\left(\frac{y^{\prime }-y}{x^{\prime }-x}\right)-\tan^{-1}\left(\frac{\left(y^{\prime }-y\right)z}{\left(x^{\prime }-x\right)r}\right)\right)+\cdots \\ & 3\left(x^{\prime }-x\right)\log\left(y^{\prime }-y+r\right) \end{array}]|}_{a_{2}}^{a_{1}}|}_{b_{2}}^{b_{1}}+\cdots \\ & \;+x\frac{\partial^{3}{\Gamma \;}^{00}}{\partial x^{3}}+3\frac{\partial^{2}{\Gamma \;}^{00}}{\partial x^{2}} \end{array}$$

$$\begin{array}{rl} \frac{\partial^{2}{\Gamma \;}^{10}}{\partial x\partial y} & ={{[\left(x^{\prime }-x\right)\left(z\log\left(z+r\right)-r\right)]|}_{a_{2}}^{a_{1}}|}_{b_{2}}^{b_{1}}+x\frac{\partial^{2}{\Gamma \;}^{00}}{\partial x\partial y}+\frac{\partial {\Gamma \;}^{00}}{\partial y}\\ \frac{\partial^{3}{\Gamma \;}^{10}}{\partial x^{2}\partial y} & ={{[r+\frac{{\left(x^{\prime }-x\right)}^{2}}{z+r}-z\log\left(z+r\right)]|}_{a_{2}}^{a_{1}}|}_{b_{2}}^{b_{1}}+x\frac{\partial^{3}{\Gamma \;}^{00}}{\partial x^{2}\partial y}+2\frac{\partial^{2}{\Gamma \;}^{00}}{\partial x\partial y} \end{array}$$

**A.3. y-Linear traction solutions**

$$\begin{array}{rl} & \frac{\partial {\text{A}}^{01}}{\partial x}={{[-r]|}_{a_{2}}^{a_{1}}|}_{b_{2}}^{b_{1}}+y\frac{\partial {\text{A}}^{00}}{\partial x}\\ & \frac{\partial^{2}{\text{A}}^{01}}{\partial x^{2}}={{[\frac{\left(x^{\prime }-x\right)}{\sqrt{{\left(x^{\prime }-x\right)}^{2}+{\left(y^{\prime }-y\right)}^{2}+z^{2}}}]|}_{a_{2}}^{a_{1}}|}_{b_{2}}^{b_{1}}+y\frac{\partial^{2}{\text{A}}^{00}}{\partial x^{2}}\\ & \frac{\partial^{2}{\text{A}}^{01}}{\partial x\partial z}={{[-\frac{z}{r}]|}_{a_{2}}^{a_{1}}|}_{b_{2}}^{b_{1}}+y\frac{\partial^{2}{\text{A}}^{00}}{\partial x\partial z} \end{array}$$

$$\begin{array}{rl} & \frac{\partial {\text{B}}^{01}}{\partial x}={{[-\frac{1}{2}\left(zr+\left({\left(x^{\prime }-x\right)}^{2}+{\left(y^{\prime }-y\right)}^{2}\right)\log\left(z+r\right)\right)]|}_{a_{2}}^{a_{1}}|}_{b_{2}}^{b_{1}}+y\frac{\partial {\text{B}}^{00}}{\partial x}\\ & \frac{\partial^{2}{\text{B}}^{01}}{\partial x^{2}}={{[\left(x^{\prime }-x\right)\log\left(z+r\right)]|}_{a_{2}}^{a_{1}}|}_{b_{2}}^{b_{1}}+y\frac{\partial^{2}{\text{B}}^{00}}{\partial x^{2}}\\ & \frac{\partial^{3}{\text{B}}^{01}}{\partial x^{3}}={{[-\frac{{\left(x^{\prime }-x\right)}^{2}}{r\left(z+r\right)}-\log\left(z+r\right)]|}_{a_{2}}^{a_{1}}|}_{b_{2}}^{b_{1}}+y\frac{\partial^{3}{\text{B}}^{00}}{\partial x^{3}} \end{array}$$

$$\begin{array}{rl} & \frac{\partial^{3}{\text{B}}^{01}}{\partial x^{2}\partial y}={{[-\frac{\left(x^{\prime }-x\right)\left(y^{\prime }-y\right)}{r\left(z+r\right)}]|}_{a_{2}}^{a_{1}}|}_{b_{2}}^{b_{1}}+y\frac{\partial^{3}{\text{B}}^{00}}{\partial x^{2}\partial y}+\frac{\partial^{2}{\text{B}}^{00}}{\partial x^{2}}\\ & {{\frac{\partial {\Gamma \;}^{01}}{\partial x}=\left[-\frac{1}{2}z^{2}r+\frac{1}{4}zr^{2}+\frac{1}{3}r^{3}-\left({\left(x^{\prime }-x\right)}^{2}+{\left(y^{\prime }-y\right)}^{2}\right)z\log\left(z+r\right)\right]|}_{a_{2}}^{a_{1}}|}_{b_{2}}^{b_{1}}+y\frac{\partial {\Gamma \;}^{00}}{\partial x}\\ & {{\;\frac{\partial^{2}{\Gamma \;}^{01}}{\partial x^{2}}=\left[\left(x^{\prime }-x\right)\left(z\log\left(z+r\right)-r\right)\right]|}_{a_{2}}^{a_{1}}|}_{b_{2}}^{b_{1}}+y\frac{\partial^{2}{\Gamma \;}^{00}}{\partial x^{2}} \end{array}$$

$$\begin{array}{rl} & \frac{\partial^{3}{\Gamma \;}^{01}}{\partial x^{3}}={{[r-z\log\left(z+r\right)+\frac{{\left(x^{\prime }-x\right)}^{2}}{z+r}]|}_{a_{2}}^{a_{1}}|}_{b_{2}}^{b_{1}}+y\frac{\partial^{3}{\Gamma \;}^{00}}{\partial x^{3}}\\ & \frac{\partial^{3}{\Gamma \;}^{01}}{\partial x^{2}\partial y}={{[\frac{(x^{\prime }-x)\left(y^{\prime }-y\right)}{z+r}]|}_{a_{2}}^{a_{1}}|}_{b_{2}}^{b_{1}}+y\frac{\partial^{3}{\Gamma \;}^{00}}{\partial x^{2}\partial y}+\frac{\partial^{2}{\Gamma \;}^{00}}{\partial x^{2}} \end{array}$$

**A.4. Bilinear traction solutions**

$$\begin{array}{rl} {\text{A}}^{11} & ={{[\frac{1}{3}{\left({\left(x^{\prime }-x\right)}^{2}+{\left(y^{\prime }-y\right)}^{2}+z^{2}\right)}^{3/2}]|}_{a_{2}}^{a_{1}}|}_{b_{2}}^{b_{1}}+x{\text{A}}^{01}+y{\text{A}}^{10}-xy{\text{A}}^{00}\\ \frac{\partial {\text{A}}^{11}}{\partial x} & ={{[-\left(x^{\prime }-x\right)r]|}_{a_{2}}^{a_{1}}|}_{b_{2}}^{b_{1}}+y\frac{\partial {\text{A}}^{10}}{\partial x}+x\frac{\partial {\text{A}}^{01}}{\partial x}+{\text{A}}^{01}-xy\frac{\partial {\text{A}}^{00}}{\partial x}-y{\text{A}}^{00}\\ \frac{\partial^{2}{\text{A}}^{11}}{\partial x^{2}} & ={{[\frac{r^{2}+{\left(x^{\prime }-x\right)}^{2}}{r}]|}_{a_{2}}^{a_{1}}|}_{b_{2}}^{b_{1}}+x\frac{\partial^{2}{\text{A}}^{01}}{\partial x^{2}}+2\frac{\partial {\text{A}}^{01}}{\partial x}+y\frac{\partial^{2}{\text{A}}^{10}}{\partial x^{2}}-xy\frac{\partial^{2}{\text{A}}^{00}}{\partial x^{2}}-2y\frac{\partial {\text{A}}^{00}}{\partial x} \end{array}$$

$$\begin{array}{rl} \frac{\partial {\text{A}}^{11}}{\partial z} & ={{[zr]|}_{a_{2}}^{a_{1}}|}_{b_{2}}^{b_{1}}+x\frac{\partial {\text{A}}^{01}}{\partial z}+y\frac{\partial {\text{A}}^{10}}{\partial z}-xy\frac{\partial {\text{A}}^{00}}{\partial z}\\ \frac{\partial^{2}{\text{A}}^{11}}{\partial x\partial z} & ={{[-\frac{\left(x^{\prime }-x\right)z}{r}]|}_{a_{2}}^{a_{1}}|}_{b_{2}}^{b_{1}}+x\frac{\partial^{2}{\text{A}}^{01}}{\partial x\partial z}+\frac{\partial {\text{A}}^{01}}{\partial z}+y\frac{\partial^{2}{\text{A}}^{10}}{\partial x\partial z}-xy\frac{\partial^{2}{\text{A}}^{00}}{\partial x\partial z}-y\frac{\partial {\text{A}}^{00}}{\partial z}\\ \frac{\partial^{2}{\text{A}}^{11}}{\partial x\partial y} & ={{[\frac{\left(x^{\prime }-x\right)\left(y^{\prime }-y\right)}{r}]|}_{a_{2}}^{a_{1}}|}_{b_{2}}^{b_{1}}+y\frac{\partial^{2}{\text{A}}^{10}}{\partial x\partial y}+\frac{\partial {\text{A}}^{10}}{\partial x}+x\frac{\partial^{2}{\text{A}}^{01}}{\partial x\partial y}+\frac{\partial {\text{A}}^{01}}{\partial y}+\cdots \\ & \;-xy\frac{\partial^{2}{\text{A}}^{00}}{\partial x\partial y}-x\frac{\partial {\text{A}}^{00}}{\partial x}-y\frac{\partial {\text{A}}^{00}}{\partial y}-{\text{A}}^{00} \end{array}$$

$$\begin{array}{rl} \frac{\partial {\text{B}}^{11}}{\partial x} & ={{[-\frac{1}{2}\left(x^{\prime }-x\right)\left(zr+\left({\left(x^{\prime }-x\right)}^{2}+{\left(y^{\prime }-y\right)}^{2}\right)\log\left(z+r\right)\right)]|}_{a_{2}}^{a_{1}}|}_{b_{2}}^{b_{1}}+\cdots \\ & \;+y\frac{\partial {\text{B}}^{10}}{\partial x}+x\frac{\partial {\text{B}}^{01}}{\partial x}+{\text{B}}^{01}-xy\frac{\partial {\text{B}}^{00}}{\partial x}-y{\text{B}}^{00}\\ \frac{\partial^{2}{\text{B}}^{11}}{\partial x^{2}} & ={{[\frac{1}{2}zr+\frac{1}{2}\left(3{\left(x^{\prime }-x\right)}^{2}+{\left(y^{\prime }-y\right)}^{2}\right)\log\left(z+r\right)]|}_{a_{2}}^{a_{1}}|}_{b_{2}}^{b_{1}}+x\frac{\partial^{2}{\text{B}}^{01}}{\partial x^{2}}+\cdots \\ & \;+2\frac{\partial {\text{B}}^{01}}{\partial x}+y\frac{\partial^{2}{\text{B}}^{10}}{\partial x^{2}}-xy\frac{\partial^{2}{\text{B}}^{00}}{\partial x^{2}}-2y\frac{\partial {\text{B}}^{00}}{\partial x} \end{array}$$

$$\begin{array}{rl} \frac{\partial^{3}{\text{B}}^{11}}{\partial x^{3}} & ={{[\begin{array}{cc} & \;-\frac{1}{2}\frac{\left(x^{\prime }-x\right)\left(3{\left(x^{\prime }-x\right)}^{2}+{\left(y^{\prime }-y\right)}^{2}\right)}{r\left(r+z\right)}+\cdots \\ & \;-\frac{1}{2}\frac{\left(x^{\prime }-x\right)z}{r}-3\left(x^{\prime }-x\right)\log\left(z+r\right)+ \end{array}]|}_{a_{2}}^{a_{1}}|}_{b_{2}}^{b_{1}}+\cdots \\ & \;+x\frac{\partial^{3}{\text{B}}^{01}}{\partial x^{3}}+3\frac{\partial^{2}{\text{B}}^{01}}{\partial x^{2}}+y\frac{\partial^{3}{\text{B}}^{10}}{\partial x^{3}}-xy\frac{\partial^{3}{\text{B}}^{00}}{\partial x^{3}}-3y\frac{\partial^{2}{\text{B}}^{00}}{\partial x^{2}}\\ \frac{\partial^{2}{\text{B}}^{11}}{\partial x\partial y} & ={{[\left(x^{\prime }-x\right)\left(y^{\prime }-y\right)\log\left(z+r\right)]|}_{a_{2}}^{a_{1}}|}_{b_{2}}^{b_{1}}+y\frac{\partial^{2}{\text{B}}^{10}}{\partial x\partial y}+\frac{\partial {\text{B}}^{10}}{\partial x}+x\frac{\partial^{2}{\text{B}}^{01}}{\partial x\partial y}+\cdots \\ & \;+\frac{\partial {\text{B}}^{01}}{\partial y}-xy\frac{\partial^{2}{\text{B}}^{00}}{\partial x\partial y}-x\frac{\partial {\text{B}}^{00}}{\partial x}-y\frac{\partial {\text{B}}^{00}}{\partial y}-{\text{B}}^{00} \end{array}$$

$$\begin{array}{rl} \frac{\partial^{3}{\text{B}}^{11}}{\partial x^{2}\partial y} & ={{[-\left(y^{\prime }-y\right)\left(\frac{{\left(x^{\prime }-x\right)}^{2}}{r\left(z+r\right)}+\log\left(z+r\right)\right)]|}_{a_{2}}^{a_{1}}|}_{b_{2}}^{b_{1}}+\cdots \\ & \;+x\frac{\partial^{3}{\text{B}}^{01}}{\partial x^{2}\partial y}+2\frac{\partial^{2}{\text{B}}^{01}}{\partial x\partial y}+y\frac{\partial^{3}{\text{B}}^{10}}{\partial x^{2}\partial y}+\frac{\partial^{2}{\text{B}}^{10}}{\partial x^{2}}+-xy\frac{\partial^{3}{\text{B}}^{00}}{\partial x^{2}\partial y}-x\frac{\partial {\text{B}}^{00}}{\partial x^{2}}-2y\frac{\partial^{2}{\text{B}}^{00}}{\partial x\partial y}-2\frac{\partial {\text{B}}^{00}}{\partial x} \end{array}$$

$$\begin{array}{rl} \frac{\partial^{2}{\Gamma \;}^{11}}{\partial x^{2}} & ={{[\begin{array}{cc} & \;\frac{1}{2}\left(z^{2}-2{\left(x^{\prime }-x\right)}^{2}\right)r-\frac{1}{4}zr^{2}-\frac{1}{3}r^{3}+\cdots \\ & \;\frac{1}{2}\left(3{\left(x^{\prime }-x\right)}^{2}+{\left(y^{\prime }-y\right)}^{2}\right)z\log\left(z+r\right) \end{array}]|}_{a_{2}}^{a_{1}}|}_{b_{2}}^{b_{1}}+\cdots \\ & \;+x\frac{\partial^{2}{\Gamma \;}^{01}}{\partial x^{2}}+2\frac{\partial {\Gamma \;}^{01}}{\partial x}+y\frac{\partial^{2}{\Gamma \;}^{10}}{\partial x^{2}}-xy\frac{\partial^{2}{\Gamma \;}^{00}}{\partial x^{2}}-2y\frac{\partial {\Gamma \;}^{00}}{\partial x} \end{array}$$

$$\begin{array}{rl} \frac{\partial^{3}{\Gamma \;}^{11}}{\partial x^{3}} & ={{[\left(x^{\prime }-x\right)\left(\frac{4{\left(x^{\prime }-x\right)}^{2}+3\left({\left(y^{\prime }-y\right)}^{2}+z\left(z+r\right)\right)}{z+r}-3z\log\left(z+r\right)\right)]|}_{a_{2}}^{a_{1}}|}_{b_{2}}^{b_{1}}+\cdots \\ & \;+x\frac{\partial^{3}{\Gamma \;}^{01}}{\partial x^{3}}+3\frac{\partial^{2}{\Gamma \;}^{01}}{\partial x^{2}}+\cdots y\frac{\partial^{3}{\Gamma \;}^{10}}{\partial x^{3}}-xy\frac{\partial^{3}{\Gamma \;}^{00}}{\partial x^{3}}-3y\frac{\partial^{2}{\Gamma \;}^{00}}{\partial x^{2}} \end{array}$$

$$\begin{array}{rl} \frac{\partial^{2}{\Gamma \;}^{11}}{\partial x\partial y} & ={{[\left(x^{\prime }-x\right)\left(y^{\prime }-y\right)\left(z\log\left(z+r\right)-r\right)]|}_{a_{2}}^{a_{1}}|}_{b_{2}}^{b_{1}}+x\frac{\partial^{2}{\Gamma \;}^{01}}{\partial x\partial y}+\frac{\partial {\Gamma \;}^{01}}{\partial y}+y\frac{\partial^{2}{\Gamma \;}^{10}}{\partial x\partial y}+\frac{\partial {\Gamma \;}^{10}}{\partial x}+\cdots \\ & \;-xy\frac{\partial^{2}{\Gamma \;}^{00}}{\partial x\partial y}-x\frac{\partial {\Gamma \;}^{00}}{\partial x}-y\frac{\partial {\Gamma \;}^{00}}{\partial y}-{\Gamma \;}^{00} \end{array}$$

$$\begin{array}{rl} \frac{\partial^{3}{\Gamma \;}^{11}}{\partial x^{2}\partial y} & ={{[\left(y^{\prime }-y\right)\left(\frac{{\left(x^{\prime }-x\right)}^{2}}{z+r}-\left(z\log\left(z+r\right)-r\right)\right)]|}_{a_{2}}^{a_{1}}|}_{b_{2}}^{b_{1}}+x\frac{\partial^{3}{\Gamma \;}^{01}}{\partial x^{2}\partial y}+2\frac{\partial^{2}{\Gamma \;}^{01}}{\partial x\partial y}+\cdots \\ & \;+y\frac{\partial^{3}{\Gamma \;}^{10}}{\partial x^{2}\partial y}+\frac{\partial^{2}{\Gamma \;}^{10}}{\partial x^{2}}-xy\frac{\partial^{3}{\Gamma \;}^{00}}{\partial x^{2}\partial y}-x\frac{\partial {\Gamma \;}^{00}}{\partial x^{2}}-2y\frac{\partial^{2}{\Gamma \;}^{00}}{\partial x\partial y}-2\frac{\partial {\Gamma \;}^{00}}{\partial x}. \end{array}$$
